# Snakebites in Two Rural Districts in Lao PDR: Community-Based Surveys Disclose High Incidence of an Invisible Public Health Problem

**DOI:** 10.1371/journal.pntd.0003887

**Published:** 2015-06-26

**Authors:** Inthanomchanh Vongphoumy, Panom Phongmany, Sengdao Sydala, Nouda Prasith, Ralf Reintjes, Joerg Blessmann

**Affiliations:** 1 Provincial Health Department of Savannakhet Province, Savannakhet, Lao PDR; 2 Hamburg University of Applied Science, Hamburg, Germany; 3 Bernhard Nocht Institute for Tropical Medicine, Hamburg, Germany; Institut de Recherche pour le Développement, BENIN

## Abstract

**Background:**

The Lao PDR (Laos) is one of the least developed countries in Asia with an estimated 25% of the population living in poverty. It is the habitat of some highly venomous snakes and the majority of the population earns their living from agricultural activities. Under these circumstances the incidence of snakebites is expected to be high.

**Methods:**

Two cross-sectional, community-based surveys were performed in Champone and Phin district, Savannakhet province, Lao PDR to estimate snakebite incidence. Multistage random sampling was used. In the first stage approximately 40% of all villages in each district were randomly selected. In the second stage 33% of all households in each village were randomly chosen. Members of the selected households were interviewed about snakebites during the previous 12 months.

**Results:**

Thirty-five of 9856 interviewees reported a snakebite in a 12 month period in Champone district and 79 of 7150 interviewees in Phin district. The estimated incidence is 355 snakebites per 100,000 persons per year and 1105 per 100,000 in Champone and Phin district respectively. All snakebite victims received treatment by traditional healers or self-treatment at home and nobody went to a hospital. Incidence of snakebites, calculated on the basis of hospital records of 14 district hospitals and Savannakhet provincial hospital, ranged from 3 to 14 cases per 100,000 persons per year between 2012 and 2014.

**Conclusion:**

Incidence of snakebites is high in rural communities in Laos with significant regional differences. Poverty most likely contributes significantly to the higher number of snakebites in Phin district. Hospital statistics profoundly underestimates snakebite incidence, because the majority of snakebite victims receive only treatment by traditional healers or self-treatment in their village. There is an urgent need to train medical staff and students in management of snakebite patients and make snake antivenom available to cope effectively with this important public health problem in order to prevent fatalities and disabilities.

## Author Summary

The World Health Organization recognized snakebites as a neglected tropical disease in 2009. Venomous snakebites cause substantial morbidity and mortality particularly in tropical developing countries and predominantly affect people in remote and poor communities with little voice and representation. Reliable epidemiological data on incidence are not available for many regions where snakebites are an important public health problem. Hospital statistics profoundly underestimate the real incidence, because the majority of the snakebite victims are treated in their village by traditional healers. Only well-designed, community-based surveys can assess the true rate of morbidity and mortality caused by snakebites in a particular area. The present surveys reveal a high incidence of snakebites in rural communities in Laos. All snakebite victims received traditional treatment in their village, because effective treatment in the hospitals is not available so far. In order to prevent disabilities and fatalities, medical staff and students need training in the management of venomous snakebites and antivenom the essential drug and only specific treatment must be available.

## Introduction

Due to subtropical and tropical climate, Laos has a rich snake fauna with 101 non-venomous and 23 venomous species described to date [1], personal communication with Alexandre Teynié]. According to surveys in Thailand and preliminary experience in Laos, six snakes within the 23 venomous species are responsible for the majority of venomous snakebites [2,3,4]. They are considered as medically important venomous snakes and snake antivenom is available for each species at the Thai Red Cross Society. Envenoming caused by each of these six snakes results in significant morbidity and mortality [5,6,7]. Two pit vipers, the Malayan pit viper (*Calloselasma rhodostoma*) and the green pit viper (*Trimeresurus albolabris*) cause severe local cytotoxic damage with swelling, blistering and necrosis, and procoagulants in the venom induce severe coagulation disorder [6]. Pre- and postsynaptic neurotoxins in the venom of 4 elapid snakes, the monocled cobra (*Naja kaouthia*), the king cobra (*Ophiophagus hannah*) the Malayan krait (*Bungarus candidus*) and banded krait (*Bungarus fasciatus*) cause muscle paralysis and death from respiratory failure, if appropriate medical care is not available. Cytotoxic components and cardiotoxin in the venom of cobras contribute to morbidity and mortality by causing severe local cutaneous and subcutaneous tissue necrosis, cardiac arrhythmia, arterial hyper- and hypotension and heart failure [7,8].

Snakebites predominantly affect poor people living in remote areas of tropical or subtropical low-income countries. They usually have very limited access to health care and particularly snake antivenom. Hence most of the snakebite victims are treated in their village by traditional healers and they won’t appear in any hospital statistics. Three major articles on worldwide snakebite epidemiology mostly rely on hospital statistics and mortality statistics of public health institutions and the authors acknowledge the underestimation of the real incidence of snakebites. In the 1950s Swaroop & Grab estimated 30,000 to 40,000 deaths from snakebite per year worldwide, excluding China, USSR and Central Europe [9]. In the 1990s Chippaux estimated an annual incidence of snakebites in Asia of 114 per 100,000 persons, a mortality rate of 2.9/100,000 and approximately 125,000 annual deaths from snakebite worldwide [10].

More recently Kasturiratne calculated an incidence of snakebite envenoming of 18–80/100,000 persons with a mortality rate of 0.1–3.1/100,000 for Southeast Asia and 20,000–94,000 death annually worldwide. The wide range of estimates reflects uncertainty [11].

Well-designed, community-based epidemiological studies are the only reliable way to assess the true rates of morbidity and mortality caused by snakebites in a particular area. They are independent of institutional reporting and it is not surprising that these surveys found much higher numbers.

A recently published nation wide household survey in Bangladesh estimated an incidence of 623 snakebites per 100,000 persons per year [12]. A community-based survey from the Eastern Terai in Nepal estimated an annual incidence of 1162 snakebites per 100,000 persons and a very high mortality of 162 deaths per 100,000 persons per year [13]. A national survey on snakebite mortality in India conducted by Mohaparta et al. found 45,900 snakebite deaths annually corresponding to a mortality rate of 4.1 deaths per 100,000 persons in India [14].

Laos still belongs to the least developed countries in the world with an estimated 25% of the population living in poverty. It is habitat of some highly venomous snakes and the majority of the population earns their living from forestry and agricultural activities. Under these circumstances incidence of snakebites is expected to be high, but no data are available so far.

Two cross-sectional, community-based surveys presented here provide epidemiological data on snakebite incidence in two different districts of Savannakhet province, Lao PDR in order to estimate the magnitude of this largely invisible and unheard public health problem in this region.

## Methods

### Study site and study population

Two cross-sectional, community-based surveys were carried out from May to August 2013 in Champone district and from March to June 2014 in Phin district in order to estimate the annual incidence of snakebites. Both districts are located in Savannakhet province in the southern part of Laos at a latitude of 16° to 17° north. Savannakhet is the largest province in Laos with a surface of approximately 21,800 km^2^, a population of 920,000 and a population density of 42 inhabitants per square Kilometre, which is higher than the average population density in Laos of 28 per km^2^. The Mekong river plain covers approximately two third of the province surface area in the west and the Annamite mountains form the border to Vietnam in the east [15,16].

Champone district is located 50 km east of the provincial capital Savannakhet in the lowland Mekong river plain. The district covers a total of 1030 km^2^ with a population density of 105 inhabitants per km^2^. The majority of the population is working in agriculture. Approximately 333 km^2^ (32%) of the district is covered with forest and 697 km^2^ (68%) is predominantly used for rice cultivation. The district has 162 villages with approximately 17,600 households and 108,000 inhabitants.

Phin district is located 160 km east of the provincial capital Savannakhet. It is almost entirely covered with forest and most parts are mountainous with altitudes up to 1000 meter. The district covers a surface of 3434 km^2^ with a low population density of 16 inhabitants per km^2^. Phin district has 144 villages with approximately 10,300 households and 56,000 inhabitants.

Beside a different geography and population density, the wealth of the inhabitants distinguishes the two districts. Individual poverty line in Laos is defined as living with less than 0.78 USD per day. A village is considered poor if more than 50% of its households are living under the individual poverty line. According to this definition no village is considered poor in Champone district, but 44% of villages in Phin district meet the criteria and the district has been identified as a poor district [17,18].

### Estimation of the population sample size

The sample size for the survey was calculated with 4 variables, *Z* for confidence level, *P* for expected proportion of snakebite victims, *d* for precision and *deff* for design effect.

The number of snakebite incidents *P* per year was estimated to be 300 snakebites per 100,000 persons. (P = 0.3%) for Champone district. This estimation was based on available data from other Asian countries, as there are neither data on snakebite incidence from Laos nor from the neighbouring countries Vietnam and Cambodia [10,12,19].

Precision d was determined as d = 0.15 and a design effect (deff) of 2 has been applied to the calculation, because cluster sampling instead of simple random sampling was used. With a confidence level of 95% (Z = 1.96) the estimated sample size was 9,753 persons. Based on the results from Champone district an incidence of 400/100,000 (P = 0.4%) was hypothesized for Phin district with a precision d = 0.2. With the same confidence level and design effect, the estimated sample size for a significant statement was 7,163 persons. Free and open source software for epidemiologic statistics has been applied for sample size calculation [20].

### Random selection of the study population

Multistage random sampling was used. In the first stage approximately 40% of all villages in each district were randomly selected (61 out of 162 villages in Champone district and 55 out of 144 villages in Phin district). In the second stage approximately 33% of all households in each village were randomly chosen. Household members were asked for a snakebite incident in the last 12 months. Research randomizer was applied to generate random numbers [21].

### Household and snakebite victim questionnaires

The survey team visited 1794 households in Champone district between May and August 2013 and 1143 households in Phin district between March and June 2014. All household members actually living in each household during the last year were listed by age and gender. After informed oral consent had been obtained from the chief of household or if not present from another adult household member, he or she was asked about an incident of snakebite within the family in the last 12 months regardless whether it was a bite from a venomous or non-venomous snake. Events like Lao New Year in mid of April or the start of Buddhist lent in July were used to make sure that the reported snakebite happened within the last 12 months. Recall bias for a snakebite is negligible. An observation period of one year was chosen, because for longer periods over several years memory of the exact year in which the snakebite happened becomes less reliable and data more imprecise. More information about circumstances of the snakebite, snake species, symptoms after the bite, what kind of treatment had been done after the bite, in which month of the year the bite happened and the outcome were obtained from those household members, who reported a snakebite. All data from questionnaires were analysed anonymously.

### Allocation of villages into forest and agriculture villages in Champone district

Sixty-one randomly selected villages in Champone district were divided into forest and agricultural villages. According to a map of Champone district designed by the World Health Organization in 2003, 29 villages were classified as forest villages and 32 villages as agricultural villages. Forest villages are located in the east and west of the district stretching from north to south and agriculture villages in the central stretch. The correct allocation of the villages was confirmed again during the visit as the map had been designed 10 years earlier. In Phin district all villages were classified as forest villages.

### Estimation of snakebite incidence from hospital statistics

In a second approach to estimate the incidence of snakebites per year, hospital administration offices of all 14 district hospitals (DHs), including Champone and Phin DH and Savannakhet provincial hospital (PH) were asked to provide data on the number of snakebite patients admitted and treated in their hospital in 2012, 2013 and 2014. All hospitals in Lao PDR are using a standard format admission book and patients are supposed to be registered in this book including an admission diagnosis, irrespective of whether they are hospitalized or not. These admission books were the basis to evaluate the number of snakebite patients treated at the DHs between 2012 and 2014. The diagnosis “snakebite” was usually recorded and is unequivocal. Five of these 14 DHs including Champone and Phin DH were visited and admission books were rechecked. The PH statistics is based on monthly evaluation of patient files and data are stored in the computer. An ongoing prospective, consecutive case series on treatment of snakebite patients at Savannakhet PH included every snakebite patient admitted to the hospital between July 2013 and December 2014 and provided reliable data. These numbers were used to check data provided by the PH.

### Statistical analysis

The snakebite incidence was calculated as the number of snakebites per 100,000 persons per year, using the number of snakebite incidents found during the survey as nominator and the number of interviewees as denominator. Chi Square test was applied to compare the risk for snakebite for people living in forest or agriculture villages.

### Ethical statement

The study was approved by the National Ethics Committee for Health Research (NECHR) at the National Institute of Public Health (NIOPH) in Vientiane capital, Lao PDR. Oral informed consent for the interview was obtained from the head of each household on behalf of his family. Only the chief of household was interviewed and only his first name appeared on the interview sheet. For the remaining household members only age and sex, but no identifying names were documented. Oral informed consent was chosen, because particularly in Phin district most of the inhabitants are from ethnic groups without a written language and most of the adult population is illiterate for the Lao language. This was approved by the board of directors of Bernhard Nocht Institute, the provincial health department in Savannakhet and the examination board of Hamburg University of Applied Science.

## Results

### Age and gender distribution of the study population

The study team visited 1794 out of 1986 randomized households in Champone district and 1143 out of 1212 randomized households in Phin district. In Champone district 192 households (9.7%) and in Phin district 69 households (5.7%) were either not at home or moved out.

A total of 9856 individuals, 5025 females and 4831 males were interviewed and included in the survey in Champone district and 7150 individuals, 3590 females and 3560 males in Phin district. The median age was 23 and 19 years in Champone and Phin district respectively.

### Incidence of snakebites in Champone and Phin district

Thirty-five out of 9856 interviewees in Champone and 79 out of 7150 interviewees in Phin district reported an incident of snakebite in the last 12 months. The calculated incidence of snakebite in Champone and Phin district is 355 snakebites per 100,000 persons per year (95% CI 254–495) and 1105 per 100,000 persons per year (95% CI 886–1376) respectively.

In Champone district 3659 interviewees were from villages in forest areas and 6197 interviewees from villages in agricultural areas. Twenty-five individuals from forest villages and 10 individuals from agricultural villages reported an incident of snakebite in the last year. Snakebite incidence is significantly higher in villages located in forest areas (683/100,000, 95% CI 458–1,012) compared to villages in agricultural areas (161/100,000, 95% CI 83–301) with an odds ratio of 4.3 (95% CI 2.0–8.9).

### Age and gender distribution of snakebite victims, circumstances of the snakebite, symptoms, treatment seeking behaviour and outcome

The median age of snakebite victims in Champone and Phin district was 29 and 30 years (range 7–70 and 3–60) and the male/female ratio was 2.9 and 2.6 respectively. Most of the snakebites happened during the rainy season between May and October, but the difference was more distinct in Champone district with a ratio of 34:1 as compared to 48:31 in Phin district. The majority of bites (78%) were located at the lower limb. There was one case of disability after snakebite in Champone district and 3 cases in Phin. These disabilities were loss of a finger (1 case) or loss of finger mobility (3 cases). No death from snakebite was reported in Champone district, while in Phin district 2 out of 79 snakebite victims died after the snakebite. According to the information given by the snakebite victims, there were more venomous snakes involved in Phin district compared with Champone district, although we acknowledge that snake identification by layman is not reliable. However most of the snakebite victims in Phin district claiming a venomous snakebite reported also local or systemic symptoms, highly suggestive for envenoming.

In Phin district 43 snakebites happened in the forest, 20 in the rice field, 6 in or close to a river and 10 in the garden. In Champone district 7 snakebites happened in the forest, 20 in the rice field, 6 in or close to a river and 2 in the garden.

In both districts all snakebite victims received treatment by traditional healers or self-treatment at home. Characteristics of snakebite victims and circumstances of the bite in Champone and Phin district are outlined in Table 1.

**Table 1:** Characteristics of snakebite victims and circumstances of the bite in Champone district (n = 35) and Phin district (n = 79).

	Champone District (n = 35)	Phin District (n = 79)
**Sex**		
Male	26	57
Female	9	22
Male : female ratio	2.9 : 1	2.6 : 1
**age**		
range	7–70	3–60
median	29	30
**Identified snake**		
Non-venomous	24	3
Venomous	9	66
**Bite site**		
Lower limb	NR	56
Upper limb	NR	16
**Symptoms after bite**		
None	27	17
Local signs (swelling, blister)	5	62
Local signs and bleeding	0	5
**Seasonal distribution**		
Rainy season (May–October)	34	48
Dry season (November–April)	1	31
**Treatment after snakebite**		
Traditional healer/self-treatment	35	79
Hospital	0	0
**Outcome**		
Favorable, no sequela	34	74
Disability after snakebite	1	3
Death after snakebite	0	2

### Snake fauna in Savannakhet province

During the present surveys in Champone and Phin district the field team distributed a brochure with pictures and information about six medically important venomous snakes in Laos, Malayan pit viper, green pit viper, monocled cobra, king cobra, Malayan krait and banded krait. Most of the interviewed villagers in both districts confirmed that these snakes are found in their region. Preliminary experience gained from treatment of 126 snakebite patients at Savannakhet provincial hospital between July 2013 and May 2015 showed that approximately 90% of venomous snakebites in this province are caused by Malayan pit viper, green pit viper and cobra, including patients from Phin and Champone district. Only two patients with Malayan krait bite were admitted to the hospital in the last two years.

### Evaluation of hospital statistics in Savannakhet province

All 14 district hospitals (DHs) of Savannakhet province and the provincial hospital (PH) administration were asked to report about the number of snakebite patients treated in their hospitals in 2012, 2013 and 2014. Thirteen DHs and the PH provided the requested data. The numbers of snakebites in five DHs were rechecked and only minor differences of 1–2 cases per hospital per year were found, except for one hospital where a difference of 5 cases was found. The PH recorded 4, 2, 13 and 49 patients with snakebites in 2012, first half-year 2013, second half-year 2013 and 2014 respectively. In comparison 20 patients were included into the prospective, consecutive case series of snakebite patients at the PH in the second half-year of 2013 and 81 patients in 2014. In Champone DH 1 (1) patient was treated in 2012, 0 (1) in 2013, but data from 2014 were missing. In Phin DH 3 (1) patients were treated in 2012, 1 (1) in 2013 and 10 (10) in 2014. The number in parenthesis indicates the rechecked number.

In 13 DHs and the PH, a total of 26 (4), 36 (22) and 127 (81) snakebite patients were treated in 2012, 2013 and 2014 respectively. The number in parenthesis indicates the number of patients treated at Savannakhet PH. According to these hospital statistics the incidence of snakebites in Savannakhet province with a population of approximately 920,000 is 3, 4 and 14 per 100,000 persons per year in 2012, 2013 and 2014 respectively.

No death had been recorded at the PH and DHs and no death was documented in the rechecked admission books of 5 DHs, including Champone and Phin DH. There are no snakebite mortality data from district or provincial health departments in Savannakhet province available.

### Evaluation of knowledge about snake fauna and management of venomous snakebites of hospital staff in 5 DHs and Savannakhet PH

Training of medical staff in 5 DHs and the PH was performed between October 2013 and July 2014. A test before the workshop with 12 basic multiple choice questions about snake fauna in Laos and management of venomous snakebites revealed low level of knowledge. On average only 43% of the questions were correctly answered by medical staff before the training.

### Availability of antivenom in provincial and district hospitals

There is no antivenom available in DHs in Savannakhet province to date. Hence medical staff has no practical experience in the use of antivenom for treatment of venomous snakebites. Antivenom is available free of charge in Savannakhet PH only since July 2013.

## Discussion

**Fig 1:**
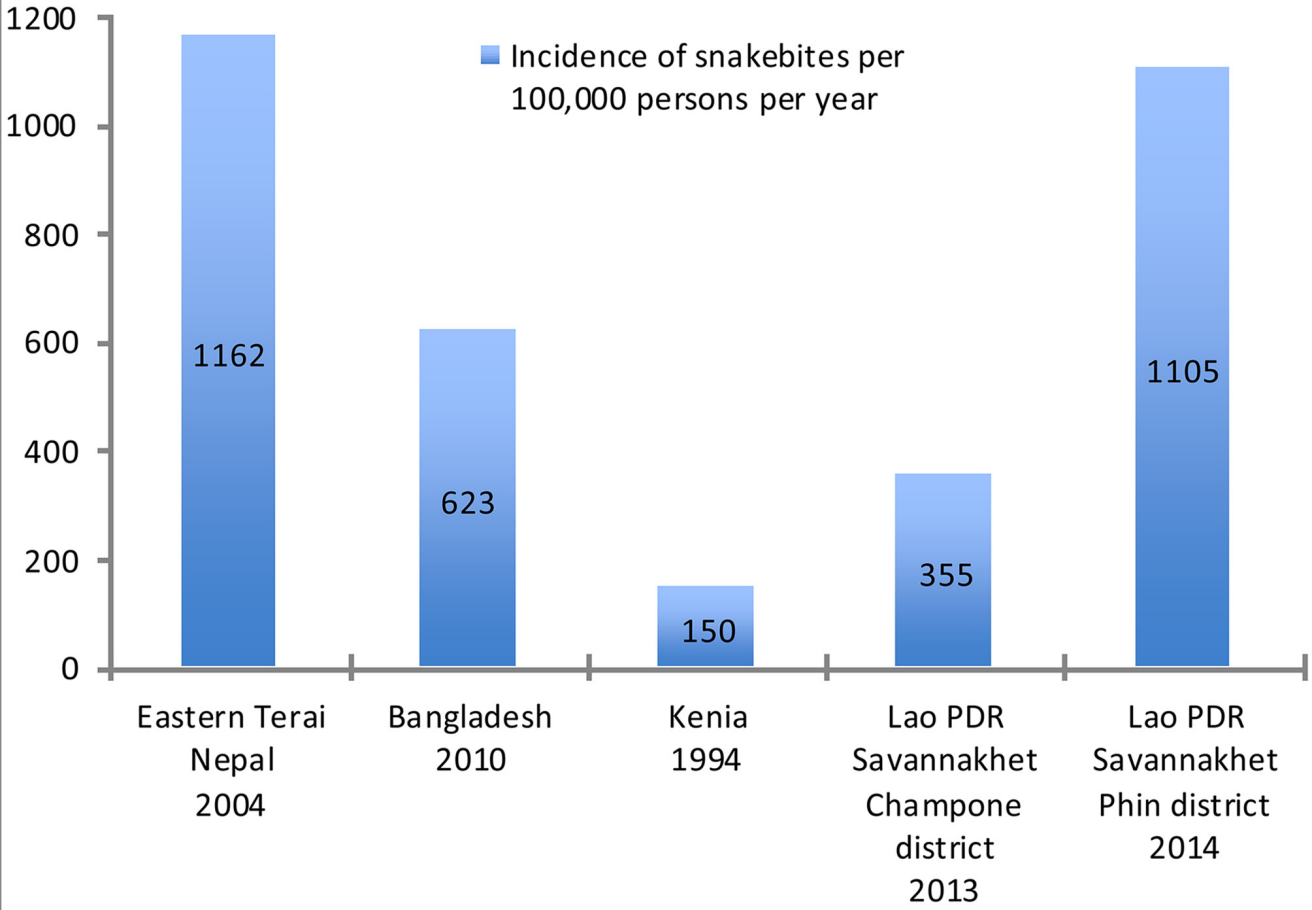
Community-based surveys on incidence of snakebites in different regions of the world.

Incidence of snakebites is high in rural communities in Laos with 355 and 1105 snakebites per 100,000 persons per year in Champone and Phin district respectively. Snakebite incidence in Phin district is one of the highest when compared with the results of other community-based surveys in different regions of the world (Fig 1) [12,22]. Only a survey in Southeastern Nepal found a similar high incidence [13]. A more than threefold higher snakebite incidence in Phin district compared to Champone, suggests significant regional differences of this public health problem in Laos.

Three major features distinguish the two survey regions. Champone district is located in the lowland Mekong river plain with two thirds of the land used for rice cultivation and only one third is covered with forest. Population density in Champone is much higher and inhabitants are wealthier. Phin district is mountainous and nearly entirely covered with forest. Population density is very low and the district was identified as a poor district with 44% poor villages according to the definition of the Lao national statistic centre [17].

In Champone district the incidence is significantly higher in forest areas, which might explain to some extent the huge difference of snakebite incidence in the two districts, because in Phin district nearly all villages are located in or close to forest areas.

Regarding the snake fauna, 124 snake species have been described in Laos to date, including 6 medically important venomous snakes, Malayan pit viper, green pit viper, monocled cobra, king cobra, Malayan krait and banded krait [1, 2, 23, 24]. Most of the interviewed villagers from Champone and Phin district confirmed that all 6 medically important venomous snakes are found in their region, but one has to acknowledge that snake identification by layman is uncertain. Preliminary experience from treatment of 126 snakebite patients at Savannakhet PH between July 2013 and May 2015 showed that 3 venomous species, Malayan pit viper, green pit viper and cobra are responsible for more than 90% of venomous snakebites in Savannakhet province. These snakes are reliably found in the province, including Champone and Phin district, possibly with varying frequency, which is difficult to verify.

Beside the potential influence of geographical differences on snakebite incidence, activities as a result of poverty like hunting animals, fishing and collecting food and wood in the forest to earn a living are most likely contributing significantly to the extremely high number of snakebites in Phin district. Wealthier people will buy their food and fish at the market and fishing becomes a recreational activity, while poor people go regularly to the forest or river to find their food free of charge with a much higher risk of snakebite.

Harrison showed a positive correlation of poverty and snakebite mortality. He acquired data on human development index (HDI), per capita expenditure on health, the percentage labour force in agriculture and gross domestic product (GDP) from databases on 138 countries for which snakebite-induced mortality rates have been estimated and each analysis illustrated a strong association between snakebite-induced mortality and poverty [25]. Rao et al. found that approximately 87% of snakebite victims with fatal outcome in Southern India lived below poverty line [26].

Snakebites predominantly affect young adult men and the majority of bites are located at the lower limbs. Most of the bites happened during the rainy season from May to October, when most villagers are working in the fields for rice cultivation. The age, sex and seasonal distribution are similar in other regions of the world [13,26,27]. In Phin district the seasonal difference is less distinct with a considerable number of snakebites during the dry season, most likely because many villagers in this area are working in the forest during that time with a high risk of snakebite.

No fatality after snakebite has been encountered in Champone district, but 2 out of 79 snakebite victims in Phin district died because of snakebite during the 12-month period. However the present surveys were designed to give evidence for incidence of snakebites and the number of interviewees is too small for a statistically significant statement on snakebite mortality. The provincial and district hospitals didn’t report any death from snakebite between 2012 and 2014, but these hospital mortality data are misleading particularly because most of the Lao people strongly believe that one should die at home, because otherwise the soul won’t find his or her native village and peace. This will have most likely considerable effect on hospital mortality statistics. The present community and hospital data do not allow any statistically reliable conclusion on snakebite mortality.

All 114 interviewed snakebite victims in both districts were treated in their village with traditional medicine and nobody went to the hospital. This explains the huge difference between the results from the community survey and the hospital statistics. Snakebite incidence varies between 3–14 per 100,000 persons per year according to data provided by the hospitals of Savannakhet province for 2012, 2013 and 2014. This profoundly underestimates the real number. Hospital statistics in developing countries are often inaccurate, but documented data of five district hospitals were rechecked and fairly accurate. However data provided by the provincial hospital underestimated the reliable number of snakebite patients obtained from the prospective, consecutive case series by approximately 40% and we cannot exclude that a certain percentage of patients at district hospitals were not recorded and are beyond our control, but it seems unlikely that the resulting underestimation would have significant impact on the huge gap between community survey and hospital data.

The fact that hospital statistics underestimate the real number of snakebites found in the community is common in many middle and low-income countries where access to health care and quality of health care are not optimal [22,28,29].

Three main reasons explain the treatment seeking behaviour in the survey region. Firstly health care utilization is often a significant financial burden and a considerable number of people cannot afford it. Secondly the strong belief in the value and benefit of traditional treatment is widespread and has been shown for snakebites in a KAP-survey performed in Champone district in 2013 [30]. According to this survey approximately 90% believe in the effectiveness of traditional medicine for snakebites. The third and most compelling reason is the fact, that snakebite patients with systemic envenoming won’t get effective treatment in the local health facilities, because knowledge of health care staff about management of venomous snakebites is not sufficient and antivenom the only effective treatment of systemic envenoming is not available in district hospitals to date. Antivenom was introduced at Savannakhet provincial hospital only in July 2013. At the same time medical staff had been trained, and district health authorities were informed about the new therapeutic options. This intervention is most likely the reason for an increasing number of snakebite patients treated at the provincial hospital in 2013 and 2014. In Phin district hospital training for management of venomous snakebites was done in January 2014 and the survey in the district was performed between March and June 2014. This probably also influenced the noticeable higher number of snakebite patients in this hospital in 2014.

The present surveys clearly indicate that venomous snakebites are a significant public health problem in Laos. Hospital statistics draw a completely different picture and snakebites don’t receive the attention they deserve. There is an urgent need to train medical staff and students in the management of snakebites and to make snake antivenom available to cope effectively with this important public health problem in order to prevent fatalities and disabilities. Health education is necessary to inform community members about first aid, treatment and prevention of snakebites.

## Supporting Information

S1 Checklist STROBE checklist. (DOC)
