# Supplementary material for: Snakebites in Two Rural Districts in Lao PDR: Community-Based Surveys Disclose High Incidence of an Invisible Public Health Problem
Source: PLoS Negl Trop Dis. 2015 Jun 26;9(6):e0003887. doi: 10.1371/journal.pntd.0003887 (PMC4482615; doi:10.1371/journal.pntd.0003887)
Supplement: S1 Checklist — (DOC) [file pntd.0003887.s001.doc]

STROBE Statement—Checklist of items that should be included in reports of ***cross-sectional studies***

|  | Item No | Recommendation |
| --- | --- | --- |
| **Title and abstract** | 1 | (*a*) Indicate the study’s design with a commonly used term in the title or the abstract  **“Community surveys” used in title and “cross-sectional community based surveys” used in the abstract. Abstract line 24-25** |
|  |  | (*b*) Provide in the abstract an informative and balanced summary of what was done and what was found. **Provided in the** **abstract line 23-47** |
| Introduction | | |
| Background/rationale | 2 | Explain the scientific background and rationale for the investigation being reported **Introduction section line 99 - 114** |
| Objectives | 3 | State specific objectives, including any prespecified hypotheses **The objective of the surveys is to provide data on snakebite incidence in two districts of Savannakhet province, Introduction section line 110 - 118** |
| Methods | | |
| Study design | 4 | Present key elements of study design early in the paper  **Methods section line 124 – 126 and line 170 – 175.** |
| Setting | 5 | Describe the setting, locations, and relevant dates, including periods of recruitment, exposure, follow-up, and data collection  **Methods section line 124 - 150** |
| Participants | 6 | (*a*) Give the eligibility criteria, and the sources and methods of selection of participants  **Methods section line 170 - 175** |
| Variables | 7 | Clearly define all outcomes, exposures, predictors, potential confounders, and effect modifiers. Give diagnostic criteria, if applicable  **Not applicable** |
| Data sources/ measurement | 8* | For each variable of interest, give sources of data and details of methods of assessment (measurement). Describe comparability of assessment methods if there is more than one group **Not applicable** |
| Bias | 9 | Describe any efforts to address potential sources of bias  **Recall bias for snakebites is negligible. Most of the people won’t forget this incident. Special dates of the year like Lao New Year in April and Start and end of the Buddhist lent in July and October have been used to make sure that the snakebite happened during the 12-month period. An observation period of one year was chosen, because for longer periods over several years memory of the exact year in which the snakebite happened becomes less reliable and data more imprecise. Methods section line 181 – 189.** |
| Study size | 10 | Explain how the study size was arrived at  **Methods section line 153- 167** |
| Quantitative variables | 11 | Explain how quantitative variables were handled in the analyses. If applicable, describe which groupings were chosen and why **Not applicable** |
| Statistical methods | 12 | (*a*) Describe all statistical methods, including those used to control for confounding  **Methods section line 222 - 226** |
|  |  | (*b*) Describe any methods used to examine subgroups and interactions  **Use of questionnaire, Method section line 189 - 193** |
|  |  | (*c*) Explain how missing data were addressed  **In both district less than 10% of households could not be visited, because people were not met at their house after several attempts or they moved out. Result section line 242 – 245** |
|  |  | (*d*) If applicable, describe analytical methods taking account of sampling strategy **Not applicable** |
|  |  | (*e*) Describe any sensitivity analyses  **Not applicable** |
| Results | | |
| Participants | 13* | (a) Report numbers of individuals at each stage of study—eg numbers potentially eligible, examined for eligibility, confirmed eligible, included in the study, completing follow-up, and analysed **Result section line 241 – 249 and table 1 for snakebite victims** |
|  |  | (b) Give reasons for non-participation at each stage **Result section line 242 - 245** |
|  |  | (c) Consider use of a flow diagram |
| Descriptive data | 14* | (a) Give characteristics of study participants (eg demographic, clinical, social) and information on exposures and potential confounders **Result section line 241 – 249 and table 1** |
|  |  | (b) Indicate number of participants with missing data for each variable of interest |
| Outcome data | 15* | Report numbers of outcome events or summary measures **result section table 1** |
| Main results | 16 | (*a*) Give unadjusted estimates and, if applicable, confounder-adjusted estimates and their precision (eg, 95% confidence interval). Make clear which confounders were adjusted for and why they were included  **Result section line 252 - 262** |
|  |  | (*b*) Report category boundaries when continuous variables were categorized **Not applicable** |
|  |  | (*c*) If relevant, consider translating estimates of relative risk into absolute risk for a meaningful time period **Not applicable** |
| Other analyses | 17 | Report other analyses done—eg analyses of subgroups and interactions, and sensitivity analyses **Result section line 266 – 285, table 1** |
| Discussion | | |
| Key results | 18 | Summarise key results with reference to study objectives  **Discussion section Line 341 - 347** |
| Limitations | 19 | Discuss limitations of the study, taking into account sources of potential bias or imprecision. Discuss both direction and magnitude of any potential bias **The study cannot provide statistically significant statements on snakebite mortality. Discussion section line 396 - 404** |
| Interpretation | 20 | Give a cautious overall interpretation of results considering objectives, limitations, multiplicity of analyses, results from similar studies, and other relevant evidence **Discussion section line 341 – 347, snakebite incidence is higher than we estimated before the surveys in both districts which gives statistically strong and significant numbers. Recall bias for snakebites is very low. Limitations are an observation period of only one year, which gives on the other hand precise results.  Methods section line 155 – 165 and line 187 - 189** |
| Generalisability | 21 | Discuss the generalisability (external validity) of the study results **The study was performed in two rural districts of Laos and the results are not representative for any other region in the country.** |
| Other information | | |
| Funding | 22 | Give the source of funding and the role of the funders for the present study and, if applicable, for the original study on which the present article is based  **The study was funded by Else Kröner-Fresenius Stiftung Bad Homburg, Germany. The funders had no role in study design, performance of the study and evaluation of the study results.** |

*Give information separately for exposed and unexposed groups.

**Note:** An Explanation and Elaboration article discusses each checklist item and gives methodological background and published examples of transparent reporting. The STROBE checklist is best used in conjunction with this article (freely available on the Web sites of PLoS Medicine at http://www.plosmedicine.org/, Annals of Internal Medicine at http://www.annals.org/, and Epidemiology at http://www.epidem.com/). Information on the STROBE Initiative is available at www.strobe-statement.org.
